# Modulation of Disordered Proteins with a Focus on Neurodegenerative Diseases and Other Pathologies

**DOI:** 10.3390/ijms20061322

**Published:** 2019-03-15

**Authors:** Anne H. S. Martinelli, Fernanda C. Lopes, Elisa B. O. John, Célia R. Carlini, Rodrigo Ligabue-Braun

**Affiliations:** 1 Department of Molecular Biology and Biotechnology & Department of Biophysics, Biosciences Institute-IB, (UFRGS), Porto Alegre CEP 91501-970, RS, Brazil; ahsmartinelli@gmail.com; 2 Center for Biotechnology, Universidade Federal do Rio Grande do Sul (UFRGS), Porto Alegre CEP 91501-970, RS, Brazil; fernandacortezlopes@gmail.com (F.C.L.); elisabeajohn@gmail.com (E.B.O.J.); 3 Graduate Program in Cell and Molecular Biology, Universidade Federal do Rio Grande do Sul (UFRGS), Porto Alegre CEP 91501-970, RS, Brazil; 4 Graduate Program in Medicine and Health Sciences, Pontifícia Universidade Católica do Rio Grande do Sul (PUCRS), Porto Alegre CEP 91410-000, RS, Brazil; 5 Brain Institute-InsCer, Laboratory of Neurotoxins, Pontifícia Universidade Católica do Rio Grande do Sul (PUCRS), Porto Alegre CEP 90610-000, RS, Brazil; 6 Department of Pharmaceutical Sciences, Universidade Federal de Ciências da Saúde de Porto Alegre (UFCSPA), Porto Alegre CEP 90050-170, RS, Brazil

**Keywords:** intrinsically disordered proteins (IDPs), neurodegenerative diseases, aggregation, drugs, drug discovery

## Abstract

Intrinsically disordered proteins (IDPs) do not have rigid 3D structures, showing changes in their folding depending on the environment or ligands. Intrinsically disordered proteins are widely spread in eukaryotic genomes, and these proteins participate in many cell regulatory metabolism processes. Some IDPs, when aberrantly folded, can be the cause of some diseases such as Alzheimer′s, Parkinson′s, and prionic, among others. In these diseases, there are modifications in parts of the protein or in its entirety. A common conformational variation of these IDPs is misfolding and aggregation, forming, for instance, neurotoxic amyloid plaques. In this review, we discuss some IDPs that are involved in neurodegenerative diseases (such as beta amyloid, alpha synuclein, tau, and the “IDP-like” PrP), cancer (p53, c-Myc), and diabetes (amylin), focusing on the structural changes of these IDPs that are linked to such pathologies. We also present the IDP modulation mechanisms that can be explored in new strategies for drug design. Lastly, we show some candidate drugs that can be used in the future for the treatment of diseases caused by misfolded IDPs, considering that cancer therapy has more advanced research in comparison to other diseases, while also discussing recent and future developments in this area of research. Therefore, we aim to provide support to the study of IDPs and their modulation mechanisms as promising approaches to combat such severe diseases.

## 1. Introduction

The protein structure–function paradigm was established in the 20th century. The key point of this paradigm is that an ordered (rigid) and unique 3D structure of a protein is an obligatory prerequisite for protein function [1,2]. Nevertheless, recent studies have provided broad and convincing evidences that some proteins do not adopt only one structure, but still are fully functional [3]. The different possible protein conformations are structured (folded), molten globular, pre-molten globular, and unstructured (unfolded) [4].

Since the beginning of the 2000s, a new class of unstructured proteins started to be studied more due to the improvement of techniques to elucidate protein structure. Crystal-structure analysis using X-ray diffraction cannot provide information on unstructured states, with only the absence of electron density in some regions being observed. However, the nuclear magnetic resonance (NMR) technique allowed for the better characterization of these disordered proteins, confirming the flexibility of protein segments that are missing in crystallography experiments [5]. They are defined mainly as intrinsically disordered proteins (IDPs), in spite of some authors defining these proteins as natively denatured [6], natively unfolded [7], intrinsically unstructured [8], and natively disordered proteins [9], among other definitions. We will use the IDP definition to refer to these proteins. The disorder could be also present in some regions of proteins; these regions are named intrinsically disordered regions (IDRs). Intrinsically disordered proteins /IDRs have no single, well-defined equilibrium structure and exist as heterogeneous ensembles of conformers [10].

**Figure 1:**
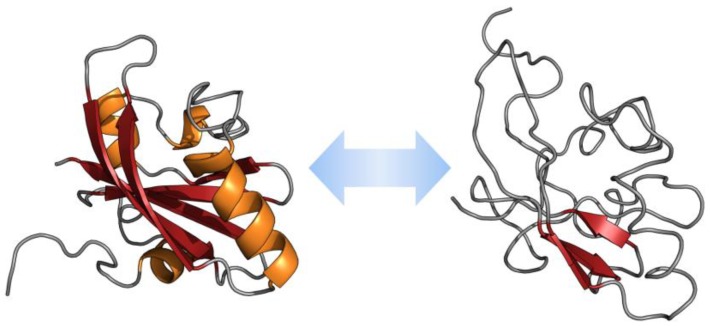
Example of intrinsically disordered proteins (IDP) conformational plasticity. Shown are ordered and disordered extremes in the conformational ensemble described for the photoactive yellow protein from *Halorhodospira halophile* (PDB IDs 3PHY and 2KX6).

There are significant differences between the amino acid sequences of IDPs/IDRs in comparison with structured globular proteins and/or domains. These differences are related to amino acid composition, sequence complexity, hydrophobicity, aromaticity, charge, flexibility, type and rate of amino acid substitutions over evolutionary time [11]. Some features of IDPs are the low content of hydrophobic residues and the high load of charged residues [12]. Intrinsically disordered proteins/IDPRs present large hydrodynamic volumes, low content of ordered secondary structure, and high structural heterogeneity. These proteins are very flexible. However, some of them show transitions from the disordered to the ordered state in the presence of natural ligands [10]. The ability of IDPs to return to the highly flexible conformations after performing their biological function, and their predisposition to acquire different conformations according to the environment, are unique properties of IDPs [13] (Figure 1).

It was demonstrated that these IDPs are highly prevalent in many genomes, including humans’, and are important in several cellular processes, such as regulation of transcription and translation, cell cycle control, and signaling [3]. It is important to highlight that they are much more common in eukaryotes, in comparison to Eubacteria and Archaea, reflecting the greater importance of disorder-associated signaling and regulation for eukaryotic cells [13]. Intrinsically disordered proteins are present in major disease pathways, such as cancer, amyloidosis, diabetes, cardiovascular, and neurodegenerative diseases. Changes in the environment and/or mutation(s) of IDPs would be expected to affect their normal function, leading to misidentification and missignaling. Consequently, it can result in misfolding and aggregation, which are known to be associated with the pathogenesis of numerous diseases. Some IDPs, such as α-synuclein, tau protein, p53, and BRCA1 are important in neurodegenerative diseases and cancer, being attractive targets for drugs modulating protein–protein interactions. Based on these IDPs and other examples, novel strategies for drug discovery have been developed [11,13]. The ability to modulate the interactions of these proteins offers tremendous opportunities of investigation in chemical biology and molecular therapeutics. Several recent small molecules, such as potential drugs, have been shown to act by blocking protein–protein interactions based on intrinsic disorder of one of the partners [14].

In this review, we will focus on IDPs involved in some neurodegenerative diseases, such as α-synuclein, amyloid β-peptide, and tau protein, while also commenting on cancer associated IDPs, such as p53 and c-Myc, and diabetes-related amylin. In addition, we will summarize the strategies to modulate IDPs action in some diseases and the promising drugs in this field, which are currently more developed for non-neurodegenerative disorders, prompting the need of focusing strategies on IDP-centered drug development for them.

## 2. Intrinsically Disordered Proteins in Some Diseases

Inside the cell, protein folding is promoted by chaperone machinery that allows the protein to adopt a folded, biologically active form [15]. However, IDPs remain partially or totally unfolded and could cause many neurodegenerative disorders due to some changes in their folding [10]. Neurodegenerative diseases are disorders characterized by progressive loss of neurons associated with deposition of proteins showing altered physicochemical properties in the brain and in peripheral organs. These proteins show misbehavior and disarrangement, affecting negatively their processing, functioning, and/or folding [16,17]. In some of these disorders, there is a conversion of the functional state of specific proteins into an aggregate state that can accumulate as fibrils, causing loss of native function, and consequent gain of a toxic function. The toxicity of these fibrils is caused by disrupting intracellular transport, overwhelming protein degradation pathways, and/or disturbing vital cell functions [16,18]. Misfolding and aggregation of IDPs/IDPRs are especially common in neurodegeneration [16,19,20].

If these misfolded proteins accumulate as deposits of aggregates, they can originate many neurodegenerative diseases such as Alzheimer’s, Parkinson’s, Huntington’s, and prionic diseases, among others [21]. Proteins that accumulate as amyloid fibrils are called amyloidogenic proteins. In order to facilitate the understanding, they can be divided in two groups: 1) proteins that present a well-defined structure with only part of the molecule being disordered, as in the case of prion protein; 2) IDPs like amyloid-β (Aβ), tau and α-synuclein, that show changes in the entire protein [22]. In addition to neurodegenerative diseases, IDPs are also involved in diabetes and different types of cancer. Here, we briefly summarize and cover the general characteristics of some IDPs that can accumulate as fibril aggregates rich in β-structure, and their association with some neurodegenerative diseases, as well as features of cancer- and diabetes-related IDPs. 

### 2.1. α-Synuclein and Parkinson’s Disease

Synucleinopathies refer to a group of neurodegenerative diseases, namely Parkinson’s disease (PD), dementia with Lewy bodies, and multiple system atrophy, characterized histologically by the presence of inclusions (Lewy bodies and Lewy neurites) composed of aggregated α-synuclein in the central nervous system (CNS) [23,24]. Aggregates containing α-synuclein can be found also in microglia and astrocytes, and in neurons of the peripheral nervous system associated with rarer autonomic diseases. This protein, encoded by the *SNCA* gene located on chromosome 4 region q21, is predominantly expressed in the brain, where it concentrates in nerve terminals. Three isoforms of synuclein, α, β, and γ, are known, but only the α isoform is found in Lewy bodies and neurites. α-synuclein is a single chain with 140 amino acids, and displays 61% identity compared to β-synuclein (134 amino acids), and its sequence contains seven imperfect repeats of eleven amino acids, each with a -KTKEGV- conserved core, separated by nine amino acid residues [25]. Weinreb and co-workers [7] reported, in 1996, the intrinsically disordered nature of α-synuclein in solution, and the protein was found to maintain its disordered state in physiological cell conditions [26]. Upon reversible binding to negatively charged phospholipids, α-synuclein oligomerizes and undergoes structural changes to assume a highly dynamic α-helical conformation while still maintaining partially disordered stretches [27,28].

The physiological role of α-synuclein is still elusive. Mice lacking all three synucleins developed only mild neurodegenerative pathology [29,30]. Lipid-bound α-synuclein accumulates in the plasma membrane of synaptic terminals and synaptic vesicles suggesting a role in neurotransmitter release [31]. The protein has been shown to possess some chaperone activity, interacting with components of the SNARE complex [32] and promoting dilatation of the exocytotic fusion pore [33]. In synucleinopathies, misfolding of lipid-bound α-synuclein occurs leading to β-sheet rich amyloid fibrils, found as the main component of Lewy bodies and Lewy neurites [20,34]. The core of fibrillated protein comprises about 70 amino acids of its repeat region, organized in parallel, in-register β-sheets in a Greek key topology [35].

In contrast to its normal, physiological form, pathological aggregated α-synuclein is extensively phosphorylated at S129 and S87. Other posttranslational modifications present in pathological α-synuclein include nitration, oxidation (for which oxidized by-products of dopamine might contribute) and truncation. Not all of these modifications contribute to accelerate the fibrillation process, since nitration and oxidation decrease fibril formation and stabilize oligomers and protofibrils of α-synuclein. On the other hand, truncated α-synuclein, typically at its C-terminal, shows increased propensity towards fibrillation. In some of the familial forms of PD, point mutations in α-synuclein (E46K, A30P, A53T) alter its propensity to fibrillate [36]. However, about 90% of Parkinson’s disease cases are idiopathic [23]. The identification of which α-synuclein species are indeed toxic is yet incomplete and is an intense field of debate. There is a growing perception that soluble oligomeric forms of α-synuclein are the most relevant in terms of toxicity, suggesting that Lewy inclusions might represent a protective response, and that interventions to favor the fibrillation process could be of therapeutic value.

Aggregation of α-synuclein apparently starts in the synapses and the aggregates propagate to nearby neurons through a prion-like mechanism [37,38]. Initial brain structures accumulating intracellular α-synuclein inclusions are the olfactory bulb, glossopharyngeal, and vagal nerves; then the Lewy pathology spreads to other regions of the brain reaching the amygdala and substantia nigra, where it causes death of dopaminergic neurons consequently leading to the motor symptoms characteristic of PD. In the more advanced cases, Lewy bodies and neurites are found in the neocortex, accounting for the cognitive impairment associated to the disease [23,39,40].

### 2.2. Amyloid β-Peptide, Tau Protein, and Alzheimer′s Disease

Alzheimer′s disease (AD) is one of the most prevalent neurodegenerative diseases that affects the learning and memory processes beyond the reduction of the brain area, degenerationand death of neurons [41,42]. Diagnosis of AD requires the identification of senile plaques composed by fibril β-amyloid peptides and tangles of tau protein aggregates [41,43]. Amyloid-β (Aβ) peptide is a well-known IDP with several oligomeric forms [44]. Amyloid-β aggregates are formed mainly by peptides containing 39 to 43 amino acids yielded by proteolytic cleavage of amyloid precursor protein (APP) [45,46,47]. Its aggregated form is significantly linked to Alzheimer′s disease, and the generation of Aβ and plaque pathology is linked to the presence of mutations or transport defects related to this protein [48,49].

The amyloid precursor protein (APP) is a transmembrane glycoprotein (type I) that is suggested to be involved in the development of the neurosystem, acting as a cell adhesion molecule [50]. The gene that encodes APP is located in human chromosome 21 [51,52] and this gene yields different isoforms by alternative splicing. Nevertheless, the function of APP is still not understood [43]. The APP proteolytic processing occurs via α, β, and γ-secretase [53]. This process can happen via two pathways: the non-amyloidogenic and the amyloidogenic route (producing toxic Aβ_1–40/42_) [54]. Aβ peptides occur in two major lengths, Aβ_1–40_ and Aβ_1–42_ amino acids, both present in senile plaques [11,46]. Some studies showed that Aβ_1–42_ accumulate as an early event in neuronal dysfunction, acting as seeding in the formation of amyloid plaques [55,56]. 

The two alloforms, Aβ_1–40_ and Aβ_1–42_, have identical sequences with the exception of two residues in the C-terminus of Aβ_1–42_, causing major differences in conformational behavior, with Aβ_1–42_ being much more folded than Aβ_1–40_ [57]. The amyloid plaques could also be associated to other molecules and metal ions, playing an important role in their assembly and toxicity [58,59]. If some mutations occur in the substrate (APP) or in the γ-secretase regulator proteins (prenisilin-1 and prenisilin-2) it may cause an alteration of APP processing, increasing the levels of Aβ_1–42_ or Aβ_1–43_ peptides formed [60,61]. These mutations are known to be involved in development of early onset AD [62,63,64,65].

In order to support the idea that Aβ peptides possess an important role in AD, Simmons and co-workers [66] demonstrated that aggregation of Aβ increased the neurotoxic effect in rat embryonic neuronal cells. Kirkitadze and co-workers [67] studied the Aβ_1–40_ and Aβ_1–42_ oligomerization and assembly into fibrils, showing that the early features of fibril assembly were the increase of intermediates containing α-helix and then their decrease by the assembly of fibrils. Yan and Wang [68] showed that Aβ_1–42_ possesses more tendencies to aggregate in comparison with Aβ_1–40_, and that their C-terminal domain is more rigid.

A structural model for amyloid Aβ_1-40_ using solid state NMR (ssNMR) spectroscopy was proposed. It was found that the first 10 residues are disordered, a β-strand conformation forming β-sheet structure was found between residues 12–24 and 30–40 [69]. After that, other studies were performed with different forms of preparation of the fibrils, with the binding of Cu^2+^, with mutant forms of the peptide, among others [70,71,72,73].

Recently, the peptide Aβ_1–42_ was studied also using ssNMR and in one of the studies it displayed triple parallel β-sheet segments, which is formed by three β-sheets encompassing residues 12–18 (β1), 24–33 (β2), and 36–40 (β3) [74]. Another NMR study of Aβ_1–42_, demonstrated that the fibril core is formed by a dimeric form of the peptide, containing four β-strands in an S-shaped amyloid fold [75]. Wälti and coworkers [76] found similar results: the fibril in dimeric form, forming a double-horseshoe. The different results of these groups were probably due to the differences in the preparation of the fibrils, such as pH, peptide concentration, agitation and ionic strength, as well as the source of the peptide (recombinant or synthetic) [42]. For a detailed review about the structural features of the two peptides, see Reference [42]. Besides the importance of Aβ_1–40_ and Aβ_1–42_, some studies demonstrated that the presence of minor isoforms of Aβ peptides could be involved in aggregation and/or or neurotoxicity [49,77,78], although their effect in AD is not fully understood.

Tau is a microtubule-associated protein initially identified as a protein involved in microtubule (MT) assembly and stabilization [79] and in the axonal transport of proteins [80]. Nowadays, the list of physiological functions of tau has expanded to include diverse roles such as protection against DNA damage and cell signaling [81]. Recent data revealed that tau physiologically interacts with various proteins and subcellular structures, and upon release from neurons, it may even act on other cells, widening the spectrum of its repercussions in health and in diseased states [82].

The single gene encoding the tau protein is present in one copy in the human genome, located in chromosome 17q21 [83,84]. Alternative splicing of this gene can yield six different isoforms of tau with polypeptide chains varying from 352 to 441 amino acids [85,86], all containing either three or four tandem repeats of 31 or 32 amino acid residues, the so-called microtubule binding repeats [81,82]. Tau is composed of 25 to 30% of charged amino acids and contains many proline residues, rendering it full intrinsically disordered. Tau undergoes many types of posttranslational modifications such as phosphorylation, glycosylation, methylation, acetylation, ubiquitinilation, SUMOylation (interaction with Small Ubiquitin-like Modifiers), nitration, among others, which are thought to finely regulate the involvement of the protein in its various biological functions. As a result of “abnormal” phosphorylation, glycosylation, oxidation, truncation or other posttranslational modification [6,82], tau becomes prone to aggregation and forms intracellular deposits, a feature of several neurodegenerative diseases collectively known as “tauopathies”. The abnormal tau adopts many transient local foldings among which β-structures of hydrophobic regions, characteristic of neurofibrillary tangles, and paired helical filaments of its microtubule binding domains [87,88] (for a review, see Reference 81]). Tau aggregates can mediate the spreading of the neuropathology to neighboring cells through its paired helical filaments, emerging as a possible target for taoupathy therapies [89]. Oxidation status of tau cysteine residues plays an important role in aggregation. While the formation of intermolecular disulfide bridges aggregates the protein, intramolecular cystine bonds prevent aggregation [90]. Truncation and/or proteolysis of tau yielding lower molecular mass forms of the protein, either in the intracellular or extracellular compartments, were also reported to lead to conformational changes that culminate in toxic, aggregated fibrillar tau [91,92].

In the most common tauopathy, Alzheimer’s disease, and in some forms of frontotemporal dementia, the sites of neurodegeneration correlate with deposits of an aberrant hyperphosphorylated tau. All six isoforms of hyperphosphorylated tau are found in tauopathies, resulting in loss of the protein’s ability to bind to microtubules and causing disturbance of axonal transport [82]. Phosphorylation of tau may occur in more than 85 putative sites, and distinct kinases and phosphatases are involved in controlling the protein’s phosphate content. On the other hand, the glycosylation and/or acetylation status of tau determines its phosphorylation pattern [93]. 

As a consequence of its disordered nature, tau interacts with a diverse array of partners inside the cell, among which are proteins, small molecules, nucleic acids, and metal ions, with many of these interactions modifying tau’s structural properties and biological functions. At least 33 distinct protein partners bind to tau’s different domains or motifs, as reviewed in Reference [81]. The multifunctionality of tau resulting from the combination of the wide range of its binding partners and a plethora of posttranslational modifications guarantees its place among true moonlighting proteins [94]. One of such interactions is with the β-amyloid peptide, in a manner that the neurotoxicity of both partners is thought to be reinforced [95,96].

### 2.3. Prion Protein in Prion Diseases

The term prion was introduced to describe a small proteinaceous agent that was causing neurodegenerative disease in humans and other animals [97]. It was identified as an abnormal form of the prion protein [98,99]. The prion protein (PrP), encoded by the *Prnp* gene, is a glycoprotein, natively found in cells and that could be involved in the maintenance of myelin in neurons among other functions [100,101,102]. Structural studies of PrP using NMR demonstrated that the N-terminal portion of the recombinant murine PrP is unstructured and flexible, and that the C-terminal portion is globular, containing 3 α-helices and a short anti-parallel β-sheet [103]. A similar structure was found for murine PrP [103], hamster PrP [104], human PrP [105] and bovine Prp [106]. Prions are not considered IDPs per se due to their mixed structural features. Some authors argue in favor of prion-specific classification [107], while others consider them to be IDP-like or IDR-containing proteins [11,108].

In prion diseases, PrP changes are predominantly from an α-helical conformation (PrPC) into a β-sheet-rich structure acquiring a PrPSc form that is misfolded, aggregated and that causes transmissible and fatal neurodegenerative diseases [18,109]. Three kinds of prion diseases have been reported: sporadic, infectious, and hereditary forms, including human disorders like Creutzfeldt–Jakob disease (CJD), Gerstmann–Sträussler–Scheinker disease (GSS), familial atypical dementia, Kuru, and veterinary disorders such as scrapie in sheep, goats, mouse, etc. [110,111]. The basic neurocytological characteristics of these diseases are a progressive vacuolation of neurons and gray matter changing to a spongiform aspect with extensive neuronal loss [112].

Interspecies transmission of prions has been postulated [113], although some interspecific barrier for transmission of PrPSc prions has been established [114]. One factor involved in this barrier could be the difference between the donor and host amino acids sequence [115,116,117]. A recent study brings new insights on prion replication during species transition [118].

The structural modifications involved in prion propagation and infectivity is the transition of α-helices of PrPc into aggregated β-sheet of PrPSC [109,119]. The presence of PrPSC abnormal form seems to stimulate and serves as template for transition of PrPC into the infectious conformation [120]. Makarava and colleagues [121] reported that prion disease could be induced in wild-type animals by injection of recombinant PrPC fibrils. In order to understand how this transition occurs, Stahl and co-workers performed a study using mass spectrometry and Edman sequencing. They demonstrated that the primary structures of PrPSc were the same as the one predicted for the *PrPC* gene, suggesting that the difference between them is not in RNA modification nor splicing events. In the same study, no covalent modifications were identified in this transition [122]. In another study, using Fourier-transform infrared (FTIR) spectroscopy and circular dichroism (CD), it was shown that PrPC contains around 42% of α-helices in its structure and only 3% of β-sheet content. On the other hand, the modified isoform PrPSc contain a higher content of β-sheet (43%) and a lower content of α-helices (30%) [109].

The PrPSc aggregates present resistance to proteolytic degradation at the C-terminal region, differently from the PrPc normal form [123]. Saverioni and co-workers [124] demonstrated that human PrPSc isolates showed strain-specific differences in their resistance to proteolytic digestion, something that could be linked to aggregate stability. Such aggregates can have heterogeneous sizes [124,125]. 

When PrPc obtains a β-sheet-rich conformation and misfolded form, it has a tendency to accumulate as amyloid fibers, a useful characteristic for detection and diagnostic of diseases [126,127]. In spite of that, the formation of amyloid plaques is not an obligatory event in prion infectivity [128]. Thinking in a therapeutic target for prion diseases, one approach would be blocking the conversion of PrPC into PrPSc [129].

### 2.4. p53, c-Myc, and Cancer

Several human diseases, such as cancer, diabetes, and autoimmune disorders, have been found to be associated with deregulation of transcription factors [130]. Carcinogenesis is a multi-step process, resulting in uncontrolled cell growth. Mutations in DNA that lead to cancer disturb these orderly processes by disrupting their regulation. This disruption results in uncontrolled cell division leading to cancer development [131]. Deregulation of multiple transcription factors has been reported in cancer progression. Extensively studied transcription factors that have shown a major role in progression of different types of cancer are p53 and c-Myc, two intrinsically disordered proteins [132,133].

Fifty percent of all human cancer present mutations in *TP53*, and on many other cancers, the function of the p53 protein is compromised. Thus, p53 is a very important target in cancer therapy [134]. Mutations in p53 are found in several types of cancer such as colon, lung, esophagus, breast, liver, brain, reticuloendothelial, and hemopoietic tissues [135]. Additionally, many p53 mutants, instead of losing functions, acquire oncogenic properties, enabling them to promote invasion, metastasis, proliferation, and cell survival [136].

p53 is a key transcription factor involved in the regulation of cell proliferation, apoptosis, DNA repair, angiogenesis, and senescence. It acts as an important defense protein against cancer onset and evolution and is negatively regulated by interaction with the oncoprotein MDM2 (murine double minute 2). In human cancers, the *TP53* gene is frequently mutated or deleted, or the wild-type p53 function is inhibited by high levels of MDM2, leading to the downregulation of tumor suppressive p53 pathways [137,138,139]. When DNA damage occurs, p53 is activated to promote the elimination or repair of the damaged cells. p53 is phosphorylated by DNA damage response (DDR) kinase, leading to cell cycle arrest, senescence, or apoptosis. In addition, p53 stimulates DNA repair by activating genes encoding components of the DNA repair machinery [140].

Human p53 is a homotetramer of 393 amino acids composed of an intrinsically disordered N-terminal transactivation domain (TAD), followed by a conserved proline-rich domain, a central and structured DNA-binding domain, and an intrinsically disordered C-terminal encoding its nuclear localization signals and oligomerization domain required for transcriptional activity [138,141,142,143,144,145,146,147]. Natively unfolded regions account for about 40% of the full-length protein and the disordered regions are extensively used to mediate and modulate interactions with other proteins. Disorder is crucial for p53 function, since its numerous posttranslational modifications are majorly found within the disordered regions [11,146,148]. The full TAD of p53 consists of the N-terminal containing 73 residues and with a net charge of −17, due to its richness in acidic amino acid residues, such as aspartic acid and glutamic acid [143]. The C-terminus, on the other hand, is rich in basic amino acids (mainly lysines) and binds DNA non-specifically [146].

Transactivation domain is a promiscuous binding site for several interacting proteins, including negative regulators as MDM2 and MDM4 [146,149,150,151]. Transactivation domain is an IDR that undergoes coupled folding and binding when interacting with partner proteins like the E3 ligase, RPA70 (the 70 kDa subunit of replication protein A) and MDM2. p53 forms an amphipathic helix when it binds to the MDM2 in a hydrophobic cleft in its N-terminal domain [137,138,149,152,153,154,155]. The p53–MDM2 interaction blocks the binding of p53 to several transcription factors. In addition, MDM2 tags p53 for ubiquitination and consequent degradation by the proteasome and the p53–MDM2 complex tends to be exported from the nucleus, preventing p53 to act as a “cellular gatekeeper” [138,144,156].

The proto-oncogene *c-MYC* encodes a transcription factor that is implicated in various cellular processes such as cell growth, proliferation, loss of differentiation and apoptosis [157]. Elevated or deregulated expression of *c-MYC* has been detected in various human cancers and is frequently associated with aggressive and poorly differentiated tumors. Some of these cancers include breast, colon, cervical, small-cell lung carcinomas, osteosarcomas, glioblastomas, melanoma, and myeloid leukemia [158,159,160]. c-Myc is a very important protein for understanding and developing therapeutics against cancers and cancer stem cells [161].

c-Myc is an IDP and becomes transcriptionally functional when it forms an heterodimer with its obligate partner Max to assume a coiled-coil structure that recognizes the E-box (enhancer-box)-sequence 5′-CACGTG-3′. The c-Myc N-terminus, its TAD, can activate transcription in mammalian cells when fused to a heterologous DNA-binding domain. The C-terminus of this protein contains a basic-helix-loop-helix-leucine zipper (b-HLH-LZ) domain, and it promotes its interaction with Max, that has the same (b-HLH-LZ) domain, and the sequence-specific DNA binding mentioned above [162,163,164,165]. Nuclear magnetic resonance studies of c-Myc disordered region have attributed to it the protein functional plasticity and multiprotein complex formation capacity [166]. Computational and experimental investigations show that c-Myc extensively employs its disorder regions to perform diverse interactions with other partners [161].

It is important to highlight that Max protein is critical for c-Myc’s transcriptional activities, both gene activation and repression [162,167]. Considering c-Myc as a target for cancer therapy, one approach to c-Myc inhibition has been to disrupt the formation of this dimeric complex [132]. However, the disruption of c-Myc-Max dimerization is not easy, since both proteins are IDPs and protein–protein interaction involving large flat surface areas are difficult to target with small molecules, such as drugs [168,169].

### 2.5. Amylin and Diabetes

Diabetes (Type II) is a multifactorial disease characterized by dysfunction of insulin action (insulin resistance) and failure of insulin secretion by pancreatic β-cells [11,170]. One hallmark feature of this disease is the accumulation of amyloid fibrils into pancreatic islets (islets of Langerhans). These amyloid deposits are majority composed by islet amyloid polypeptides (IAPP), also called amylin. Islet amyloid polypeptides are IDPs composed of 37 amino acid residues, co-secreted with insulin by the same pancreatic cells, and its gene is located on chromosome 12 in humans [171,172,173]. 

The process of aggregation of IAPP seems to be initiated by interaction of one IAPP monomer to another, progressively leading to the formation of aggregates [174,175]. Analysis of human IAPP using circular dichroism spectroscopy demonstrated that the fibril formation was accompanied by a conformational change of random coil to β-sheet/α-helical structure [176]. These transient conformations were further confirmed by other studies [177,178,179]. 

Cytotoxicity of IAPP accumulated as amyloid deposits could be associated with loss of pancreatic β-cells functions and cells apoptosis [180,181]. Recent reviews of computational studies provided mechanistic insights of IAPP structure as monomers and oligomers and their interaction with lipid bilayers in order to understand the IAPP cytotoxicity mediated by membranes [175,182].

## 3. Strategies for IDP Modulation 

Intrinsically disordered proteins can rapidly populate different conformations in solution, usually not assuming a well-defined three-dimensional structure in their native state, as a result of their signature low-sequence complexity and low proportion of bulky hydrophobic amino acids (instead, charged and hydrophilic residues are common) that lead to a flexible, dynamically disordered behavior and larger interaction surface areas than analogous folded regions in globular proteins [183,184,185,186]. Considering that IDPs participate in numerous key processes in cell metabolism, it is expected that their activity would be regulated by multiple mechanisms at transcriptional, post-transcriptional, and translational levels, which makes active IDPs accessible in shorter periods compared to structured proteins [187,188]. Some aspects of IDP modulation (mainly for IDPs involved in cell signaling) are covered in this section, arbitrarily grouped in mechanisms that engage in direct structural changes on IDPs in order to achieve stabilization (coupled folding and binding, post-translational modifications), and mechanisms that control IDPs abundance in the cell (mRNA decay, IDP proteasomal degradation, nanny model for stabilization).

### 3.1. Regulation of IDP Activity through Structural Changes

Intrinsically disordered proteins acting in intracellular pathways contain conserved motifs for interaction with nucleic acids and other proteins, and frequently form low-affinity complexes advantageous to processes like signal transduction [184]. The recognition elements (being often called “SLiMs”—short linear motifs ranging from 3 to 10 amino acids [189,190,191]) determine a pivotal feature associated with disordered proteins, that is their binding promiscuity, which is carried out through “one-to-many” and “many-to-one” mechanisms [192]. Interestingly, many IDPs are able to adopt ordered structures when interacting with certain targets, characterizing the coupled folding and binding phenomenon. Also, structural polymorphisms can emerge from the IDP conformational landscape in cases where the same disordered protein can assume different defined structures as it binds to different targets. Some of the recognition elements are also targeted for post-translational modifications by regulatory enzymes, enabling disordered-to-ordered transitions in IDPs. However, induced folding in IDPs is not mandatory for activity, as many regions that remain disordered upon partner binding are important to function, constituting “fuzzy” complexes [193,194].

#### 3.1.1. Coupled Folding and Binding

The folding induction by partner interaction is possibly one of the most reported characteristics of IDPs, despite not being a phenomenon absolutely widespread across this class of proteins (considering the fuzzy complexes), nor a completely understood process. There are numerous examples of disordered-to-ordered transitions in proteins implicated in the regulation of gene expression, like transcription factors that assume folded motifs when interacting with DNA. One particular example is the leucine zipper protein GCN4, which presents a basic region that is unstructured in the absence of DNA but becomes a stable helical structure when interacting to its cognate AP-1 site [195]. The transition begins with transient nascent helical forms, observed in the unbound state, that interact with DNA and lead to dramatic structural changes, explained by a reduction of the entropic cost of DNA binding due to restriction of the conformational space accessible to the basic region [8,196]. Thus, the induced folding is usually explained by loss of conformational entropy (from the unbound IDP state) upon target binding and compensatory favorable contributions from reduction in exposed hydrophobic surfaces and enhanced electrostatic interactions [196]. Exhaustive kinetic studies are necessary for the investigation on what order the events of binding and folding occur, and segregate them in schemes of induced fit (IF, when the IDP binds to a partner and then folds) or conformational selection (CS, when the partner only binds to IDPs in a certain conformation) [197]. However, trying to define the coupled folding and binding in two categories may offer a rather simplistic explanation for the IDPs behavior, since the mechanisms can overlap depending upon the system conditions [197,198]. 

#### 3.1.2. Post-Translational Modifications

Because of their accessibility to modifying enzymes, IDPs are frequent targets for post-translational modifications (PTMs), which expand their functional versatility [185,199]. The occurrence of PTMs causes structural changes on IDPs by affecting their energy landscapes, due to modifications of the physicochemical properties of the primary sequence [185]. Post-translational modifications engage in the addition of chemical functional groups (usually small radicals such as phosphoryl, alkyl, acyl or glycosyl) or involve the direct modification of residues through reactions of oxidation, deimidation, and deamidation. Intrinsically disordered proteins suffering PTMs may have their electrostatic, steric, and hydrophobic properties modified, possibly inducing transformations on the structure due to enhancement/inhibition of contacts of motifs within the IDP chain or with binding partners [185,200,201,202]. Phosphorylation is one of the most prevalent PTM and constitutes a major regulatory mechanism in various cellular processes involving signal transduction. Replacing a neutral hydroxyl group with a tetrahedral phosphoryl results in new possibilities for intra- and intermolecular electrostatic interactions (e.g., salt bridges and hydrogen bonds). Many other types of PTMs work in a similar fashion (but modulating different chemical properties), providing new forms of interaction that can ultimately cause alterations in IDPs activity, including disordered to ordered transitions. 

### 3.2. Regulation of IDPs Abundance

Intrinsically disordered proteins levels are carefully monitored in the cell, and changes in their abundance are associated with disease, mainly due to defective signal transduction (linked to the occurrence of some cancers [11,203]) and non-specific interactions that generate fibrillar aggregates (present in many neurodegenerative disorders [199,204]). Tight regulation can be achieved in different levels, controlling the half-lives of mRNAs encoding IDPs and the abundance of IDPs themselves. Obviously, there are outliers for these global trends and certain IDPs are present in cells in large amounts or for long periods of time [205]—usually not the IDPs involved in dynamic processes such as cell signaling. Examples include the fibrous muscle protein titin [206,207] and the curious case of tardigrade-specific IDPs, that are constitutively expressed and upregulated in some tardigrade species and are essential for desiccation tolerance [208].

#### 3.2.1. IDP-Encoding mRNAs

A robust study monitoring gene expression in *Saccharomyces cerevisiae* (with similar trends detected for human genes [205]) demonstrated that mRNAs encoding sequences categorized as “highly unstructured” have lower half-lives than mRNAs encoding more structured proteins, having a comparable number of transcription factors regulating them [206]. One of the reasons for the increased mRNA decay is hypothesized to be the short poly(A) tails observed in IDP-encoding mRNAs, that foment RNA degradation pathways. Moreover, other factors related to transcript instability, like the binding of RNA-binding PUF proteins (that usually facilitate deadenylation and subsequent RNA clearance [209,210]) were found to be increased in mRNA coding for disordered proteins [206]. 

#### 3.2.2. Proteasomal Degradation

Intrinsically disordered proteins undergo proteasomal degradation through two different (but not mutually exclusive) pathways, ubiquitin-dependent (UD) and ubiquitin-independent (UI) [211]. The UD pathway relies on the addition of ubiquitin to the substrate to be degraded, in a process regulated by a series of enzymes and is mediated collectively by the 26S proteasome [212]. It was found that IDPs present a high content of predicted ubiquitination sites [185,205], and, in an analysis of ubiquitinated proteins, there seems to be a correlation between confirmed degradation sites and regions of disorder [213]. Alternatively, the UI pathway is mainly orchestrated by the core of the 20S proteasome, being a default process for degradation of free disordered proteins. Some evidences support the hypothesis that the flexible and extended structures of IDPs (as well as disordered terminal segments in folded proteins) facilitates the interaction with the proteasome, considering that bulky particles have reduced cleavage rates [214,215].

#### 3.2.3. Stabilization through “Nanny” Proteins

As IDPs are usually prone to degradation, there is a need for protein stabilization in some contexts. The ubiquitous enzyme NQO1 functions as a “gatekeeper” of the 20S proteasomes, binding and regulating the degradation of some IDPs, in a mechanism consuming NADH [211,216]. An analogous mechanism characterizes the “nanny” model for IDP protection from cleavage, where there is sequestration of ID segments by interactions with other proteins, resulting in evasion from 20S proteasomal digestion. The binding of the nanny is transient, beginning at the initial stage of IDPs’ life cycle, when they are newly synthesized (assuming they are more sensible to digestion in this stage) [154]. Despite the fact that nanny proteins also bind to nascent polypeptide chains, they are not considered chaperones because they do not induce a fixed three-dimensional organization on targets, only assisting on IDP conservation without permanently affecting their disordered structure.

### 3.3. Modulation of IDPs by Chaperones and Co-Chaperones

Aggregates produced in neurodegenerative diseases have been shown to respond to changes in levels of molecular chaperones, suggesting the possibility of therapeutic intervention and a role for chaperones in disease pathogenesis [217]. The heat shock protein Hsp90 promotes neurodegenerative disorders indirectly [218]. Tau protein accumulation is regulated by a (Hsp90) chaperone system. This chaperone is able to bind Tau, causing a conformational change that allows tau’s phosphorylation by glycogen synthase kinase (GSK3β), leading to tau aggregation [219]. The inhibition of this chaperone results in the reduction of tau phosphorylation levels, due to reduction of GSK3β levels [220]. Another approach was performed, the use of a co-chaperone of Hsp90, ATPase homolog 1 (Aha1), this protein is an activator of Hsp90. This approach promoted the increase of the production of aggregated tau in vitro and in mouse model of neurodegenerative disease. Moreover, inhibition of Aha1 reduced tau accumulation in cultured cells. Thus, Aha1 is an interesting target to the treatment of Alzheimer’s disease [221]. 

The Hsp70 is a protein stabilizer, has a cellular protection against neurodegeneration of the central nervous system [218]. Members of the Hsp70 family, such as Hsp70 and Hsc70, bind to misfolded proteins and somehow send them to the lysosome–autophagy pathway or ubiquitin–proteasome system for degradation [222,223]. Folding and degradation of proteins are linked through co-chaperones, such as C-terminus of HSP70-interacting protein (CHIP) and HSJ1 (DNAJB2) [224] which regulate the decisions determining whether misfolded proteins are refolded or degraded. The CHIP is associated with α-synuclein inclusions and act as a co-chaperone, altering its aggregation and enhancing the degradation of the misfolded α-synuclein [225].

Another important chaperone is cyclophilin 40 (CyP40) that is a cis/trans peptidyl-prolyl isomerase (PPIase) and is involved in regulation and orientation of proline residues [226,227]. Tau protein is rich in proline residues and its residues, usually found in β-turns, are involved in tau aggregation propensity [228]. Based on this information, Baker and coworkers [229] demonstrated that CyP40 possess the ability to dissolve amyloids fibrils in vitro. Nuclear magnetic resonance experiments showed that CyP40 acts specifically on proline rich residues performing the disaggregation of tau fibrils and oligomers. This cyclophilin could also interact with others aggregated proteins containing proline, like α-synuclein [229].

## 4. Known Drugs Acting on IDPs

Despite IDPs abundance in eukaryotes, currently there are no FDA-approved drugs specifically targeting these proteins, only experimental and speculative ones (i.e. drugs that have been evaluated by the United States Food and Drug Administration agency and had their marketing sanctioned). Some experimental drug examples are prevalent in the literature, such as those targeting p53-MDM2, c-Myc-Max, and EWS-Fli1 complexes, while some others are less discussed [230,231,232,233]. In this section we provide an overview of the pharmacological modulation of IDPs, neurodegenerative and otherwise.

Intrinsically disordered proteins are normally considered aggregation-avoidant, due to their high proportion of charged residues (as opposed to patches of folding-inducing, hydrophobic residues). Such “non-folding” plasticity is proposed to be advantageous for proteins with multiple partners [234,235]. However, some of the IDPs, as described earlier, are found in conformational diseases and amyloid formation (e.g., in Alzheimer’s disease, Parkinson’s disease). The suppression of fibril formation, thus, is of therapeutic interest. Drug candidates in this front include molecular tweezers (Table 1), which are ligands designed to bind lysine and arginine specifically, perturbing aggregation [236,237,238]. These positively-charged residues are prone to interact with negatively-charged regions in the fibril-forming monomers [237]. The SEN1576 compound, a 5-aryloxypyrimidine inhibitor of synaptotoxic Aβ aggregation (Table 1) was shown to be safe and orally bioavailable with good brain penetration [239].

Fragments of amyloid fibrils also served as templates for non-natural amino acid inhibitors of amyloid fibril formation (d-TLKIVW) [240], while the ELN484228 (Table 1) compound was shown to be protective in cell models for vesicular dysfunction via α-Synuclein [235]. Alterations of the neuroleptic agent chlorpromazine allowed for enhanced 20S proteasome activation, inducing degradation of IDPs, such as tau and α-synuclein, but not of structured proteins [241]. These chlorpromazine-derived molecules, despite showing noteworthy potential (even as tools to study the proteasome 20S gate regulation), may interfere with other, physiological, non-pathologic disordered proteins to a still unstudied extent. A naphthoquinone-tryptophan hybrid (NQTrp) (Table 1) was shown to be effective in model systems for tau aggregation [242].

Regarding tumor-associated IDPs, the most commonly mutated gene in human cancers, the tumor suppressor protein p53, is key to cell cycle signaling. It is regulated by binding to various partners, including MDM2 and Taz2 [243,244,245]. Despite being highly disordered, p53 is not itself the target for the currently screened drug candidates aiming the p53-MDM2 complex. Instead, these ligands aim to occupy the p53-binding site in MDM2. The inhibitors nutlins (Table 1) (*cis*-imidazoline analogs currently in phase-I clinical trials), were shown to be potent against multiple cancerous cell lines, including breast cancer, colorectal cancer, lung cancer, osteosarcoma, prostate cancer, and renal cancer [246,247,248,249].

The c-Myc-Max complex involves the IDP transcription factor c-Myc that is activated by binding to Max, being expressed constitutively in various cancer cells [250]. Inhibitor candidates target the disordered c-Myc in this case, including peptidomimetic inhibitors [249,250], the small molecules 10058-F4, 10074-G5 (Table 1), and some others [250,251,252,253,254,255,256,257]. The oncogenic fusion protein EWS-Fli1 is an IDP exclusively present in Ewing’s sarcoma [257]. As with c-Myc-Max inhibitors, the small molecule inhibitor YK-4–279 (Table 1) targets the disordered EWS-Fli1 protein directly [257,258].

The AF9-AF4 dimer is found in acute leukemias and is composed by two disordered fusion proteins. The AF9 protein is responsible for turning hematopoietic cells oncogenic [259,260]. The AF4-derived peptide of amino acid sequence PFWT was shown to inhibit AF9 when used in combination with established chemotherapeutic agents [261,262], while some non-peptidic inhibitor candidates have been identified by high-throughput screening [263]. The protein-tyrosine phosphatase 1B (PTP1B), a reticular non-transmembrane enzyme, has been validated as therapeutic target for diabetes, obesity, and breast cancer, due to its role as negative regulator of insulin and leptin signaling [264]. Protein-tyrosine phosphatase 1B has an elongated disordered carboxy-terminus, to which trodusquemine (Table 1) (MSI-1436, a natural product) binds [265]. This aminosterol acts allosterically, stabilizing an inactive form of the enzyme by binding to a non-catalytic disordered site [265].

Inhibitors of α-synuclein aggregation are considered a promising approach. From in vitro studies, a few lead molecules were identified, such as EGCG (epigallocatechin gallate) [266]. Iron is known to induce the aggregation of α-synuclein. Deferiprone is an iron chelator used in thalassemic patients. Two clinical trials have shown a decrease in iron content in the substantia nigra of some PD patients, while a trend for improved motor scores was seen for all enrolled patients [267]. Although copper can induce aggregation of α-synuclein in vitro, levels of copper in the substantia nigra of PD patients are up to 50% lower than that of age-matched controls. The Cu^2+^ complex of diacetylbis-(4-methylthiosemicarbazone), called Cu^2+^(atsm), showed neuroprotective action in different animal models of PD [268], prompting for a phase I trial. From in vivo studies, promising results were obtained for KYP-2047, an inhibitor of prolyl oligopeptidase, an enzyme shown to interact with α-synuclein [269,270,271]; the diphenyl-pyrazole compound anle138b, was shown to cross the blood-brain barrier of mice and to reduce aggregation of α-synuclein [269]. The NPT200-11 compound prevented the formation of oligomers of α-synuclein and improved neuropathological symptoms in transgenic mice [272] and it has already been subjected to a phase I clinical trial. Another promising compound, NPT088, is a fusion protein of a general amyloid interaction motif derived from a bacteriophage [273] and a fragment of human immunoglobin, developed by Proclara Biociences, entered a phase I clinical trial for AD [274]. Phase II clinical trial of NPT088 is expected to include PD patients. Intrabodies, a single chain variable fragment of immunoglobulin expressed intracellularly, have been developed to target oligomeric and fibrillary α-synuclein, conferred neuroprotection, apparently by shifting the dynamics of the aggregation process [275,276,277]. Addition of a proteasome-addressing sequence to intrabodies targeted pathological forms of α-synuclein to degradation, NbSyn87PEST, directed towards the C-terminal region, and VH14PEST, directed against the NAC hydrophobic interaction domain, effectively degraded α-synuclein in cultured cells [278]. In rats overexpressing wild-type α-synuclein, these proteasome-targeted intrabodies (or nanobodies) decreased the levels of pathological aggregates, increased striatal dopamine levels and improved motor function [279]. Research in this promising field moves to find ways to deliver these compounds in adequate levels in specific areas of the brain, probably by using viral vectors.

Immunotherapies against α-synuclein, based on the evidence of an extracellular pathological protein during spreading of PD to different brain structures, show promising results in animal models [280]. Besides opsonization of the pathological protein for clearance, it is likely that antibodies could block further oligomerization of α-synuclein. Both passive (humanized monoclonal antibodies) and active (vaccine) immunization are being pursued. Pharmaceutical companies have joined the efforts and early clinical trials have been concluded or are under way. A brief description of the more advanced planned immunotherapies follows. A phase I trial was conducted by Roche for PRX002, a monoclonal antibody against the C-terminus of α-synuclein. It was well tolerated and reduced by 96% the levels of serum α-synuclein [281,282]. Affitope PD01A, a synthetic α-synuclein-mimicking peptide developed by Affiris for active immunization, had the first pilot study in 21 PD patients concluded in May 2018. It elicited a specific antibody response and showed good safety and tolerability profiles in a long-term (4 years) outpatient setting. Results of Affitope PD03A phase I clinical trial indicated no severe off-target effects, and a dose-dependent production of antibodies that cross-reacted with the intended α-synuclein epitope. Results from animal studies demonstrated that the antibodies raised against these antigens crossed the blood-brain barrier, decreasing the levels of aggregated α-synuclein, thereby improving motor function [283,284].

Regarding tauopathies, various therapeutic approaches have been tested, aiming to inhibit aggregation of tau, either directly or by preventing its interaction with some partners, and removal of toxic conformers and fibrillated tau [80]. However, the enormous effort put on finding ways to revert or delay the neurodegeneration symptoms associated to fibrillar tau, or to prevent the onset of tauopathies, has been so far unsuccessful, partly due to the intrinsically disordered nature of tau, which hampers drug design based on structural approaches. Small molecules that inhibit tau aggregation in vitro are considered promising leads to anti-tauopathy drugs [285] and the number of new tau inhibitory molecules grows steadily [286]. Nevertheless, there are unanswered questions regarding their effectiveness in vivo and the potential non-specific effects on normal tau physiology that could impact heavily on the CNS. The most studied small inhibitory molecules belong to distinct chemical groups, such as phenotiazines, cyanines, rhodanines, and arylmethines [287,288]. Peptides derived from neuroprotective proteins like NAP (amino acid sequence NAPVSIPQ) and d-SAL (all D-amino acid sequence SALLRSIPA) [289,290], enantiomeric peptides [291], and RNA/DNA aptamers [292] are also attractive components of future anti-tauopathy therapies. Some natural molecules present in cellular medium, such vitamin B_12_ [293] and 8-nitro-_C_GMP [294], are known to inhibit tau aggregation through oxidation of its cysteine residues. Drugs that bind tau, inducing formation of intramolecular disulfide bonds, such as methylene blue [295] or cinnamon derivatives [296], are potential frames for developing specific tau aggregation inhibitors. Taking into account that accumulation of hyperphosphorylated tau is a hallmark of AD and other neurodegenerative disorders, inhibitors of kinases, particularly of glycogen sintase kinase 3β and of Fyn, a member the Src-family of non-receptor tyrosine kinases, have drawn much attention for their anti-tauopathy potential [80]. Another strategy focuses on dual inhibitors that would interfere on tau aggregability and simultaneously block its interaction with protein partners, particularly kinases [297,298]. Other attempts to develop an anti-tauopathy drug have focused on inhibiting tau interaction with proteases like beta-secretases [299], caspases [300], and calpain [301], and chaperones such as Hsp90 [302], among others.

Tau-targeted immunotherapy began in 2013 [303], and since then a dozen of different types of immunological strategies were subject of clinical trials, including two active immunizations (vaccines) and humanized monoclonal antibodies directed towards distinct tau epitopes aiming passive immunization (reviewed in References [304,305]). These clinical trials are still at early phases and only limited data on the outcomes have been disclosed so far [306,307]. To achieve a successful immunotherapy to treat tauopathies, antibodies should be capable of neutralizing at least one of the many diseased isoforms of tau, either intracellularly or in the extracellular space, and interrupt the processes that lead to tau fibrillation and the neuron-to-neuron spreading. Ideally, the antibodies do not bind to normal tau and are able to cross the blood–brain barrier. In the case of a tau vaccine, the senescence of the immune system of the elderly has to be considered [304]. AADvac1 was conceived as the first vaccine against AD, using as immunogen a tau peptide previously identified to be essential for its pathological aggregation. Active immunization with this peptide elicits antibodies against a stretch of tau’s primary sequence (amino acid residues 294–305) and to conformational epitopes as well, targeting mainly extracellular tau, reducing its oligomerization. Tested in different animal models of AD, AADvac1 raised a protective humoral immune response with antibodies that discriminated between normal and pathological tau, reduced the level of neurofibrillary pathology in rat brains and lowered the content of disease-specific hyperphosphorylated tau [308]. Phase I clinical trial of AADvac1, conducted in 2013–2015 in patients aged 50–85 years with mild-to-moderate AD immunized weekly for 12 weeks, revealed a favorable safety profile and 29 out of 30 patients given AADvac1 developed an IgG response [309]. After 72 weeks, and booster doses of AADvac1, patients who had developed higher IgG titers showed lower hippocampal atrophy and cognitive decline rates and only mild adverse side effects [310]. A second active immunotherapy against tau has the compound ACI-35 as the immunogen, a peptide containing tau’s phospho-epitope pS396/pS404, in a liposome-based formulation able to elicit antibodies against abnormal hyperphosphorylated tau in P301L tau-mice [311]. 

Attempts of passive immunotherapy utilize humanized monoclonal antibodies, mostly of IgG1 or IgG4 isotypes, which are directed towards stretches of tau’s primary sequence known to be involved in the oligomerization of the protein, or to extracellular seeding-capable forms of truncated tau [304]. Phase I clinical trial of ABBV-8E12, one of such humanized monoclonal antibodies [312], revealed a satisfactory safety profile in 30 patients with the progressive supranuclear palsy tauopathy, receiving single doses (2.5 to 50 mg/kg) of the antibody, with no signs of immunogenicity against it [313]. Phase I trials of other two anti-tau antibodies, C2N-8E12 and BMS-986168, were also conducted [307].

Something that is noteworthy, and that demonstrates the close interplay between amyloid β peptide and tau in causing neurodegenerative diseases, therapeutic interventions aimed at one pathology can ameliorate symptoms of the other. This is the case of immunization of triple transgenic AD-like mice with a full-length DNA of amyloid β_1–42_ peptide, which showed a 40% reduction in the brain content of the amyloid β_1–42_ concomitant with a 25–50% decrease of total tau and different phosphorylated tau isoforms [314]. Conversely, passive immunization with antibodies against tau’s fragments 6–18 and 184–195 protected triple transgenic AD-like mice by reducing amyloid precursor protein in the CA1 region of hypothalamus and in amyloid plaques [315,316].

## 5. Status and Challenges in Drug Development for IDPs

The limited number of drugs targeting IDPs currently available (see previous section) may look disappointing, considering the physiological relevance of these proteins. One should be careful, though, to not exaggerate the current lack of IDP-specific drugs as being a reflection of disorder as a limitation for drug development. 

The rational drug design strategy has been used successfully since the 1980s [317,318,319]. It depends on knowledge of the three-dimensional structure of the target protein, based on which ligands (usually inhibitors) are planned with aid of computational tools [320]. By definition, IDPs do not have a single, major conformation, occurring in dynamic conformational ensembles [10], and there is difficulty in using such traditional techniques to design IDP ligands [321]. Hence, most cases of IDP drug development were carried out by experimental screening and not by rational design [322]. Still, detection of IDP “hits” (potential initial drug candidates) through high throughput screening of compounds has been challenging [321] and computational methods achieved some success in predicting good candidates [323].

For IDPs with recognizable/determined metastable structures in their conformational ensembles, such structures could be used for rational drug design. However, IDPs are expected to be promiscuous, acting as hubs for multiple cellular processes [324]. This scenario, described as “protein clouds” [325] has its complexity further increased, with IDP ligands being described as “ligand clouds around protein clouds” [326]. Such roles in protein–protein interactions (PPIs) make IDPs especially interesting as drug targets, but the development of molecules targeting PPIs has been in itself, challenging [327,328,329,330]. 

A large difference is expected between the entropic loss and the enthalpic gain upon binding of a small ligand to an IDP, but some of them were shown to be capable of forming adaptable, specific interfaces for small molecule binding [231,235,321]. Intrinsically disordered proteins are difficult targets, since their interactions with small molecules are weaker and more transient, and the entropic loss is greater, in comparison to structured proteins [331]. The fragment-based drug design approach allows fragments to sample large amounts of chemical space, reducing the number of compounds for screening, with different fragments that bind at different regions of IDPs being able to be linked together via an appropriate linker [331]. Such fragments usually require hydrogen bonds to achieve detectable binding, generating an enthalpic gain that compensates for the entropic loss upon binding of the small molecules, lowering the free energy of the protein upon binding [331].

Still, the over-representation of IDPs in disorders, as summarized by the D^2^ concept (for “disorder in disorders”) [11] and the D^3^ concept (for “disorder in degenerative disorders”) [16], points to these proteins as promising therapeutic targets. Attempts to detect potentially druggable cavities in IDPs have identified at least 14 targets that could be subjected to rational drug design [233]. A more general estimate is that 9% of detected cavities may be druggable in IDPs, in comparison to 5% in ordered proteins [233]. These observations are especially interesting, considering that current drugs target around 500 proteins, less than 10% of the estimated potential target list, with very strict classes (such as enzymes and G-protein coupled receptors) accounting for more than 70% of them [230,331,332].

There have been major advances in the detection and prediction of IDP features in protein sequences [293], something that will surely help in the identification of these special drug targets. Major breakthroughs are also being achieved by the combination of experimental methods (especially NMR and fluorescence techniques) with computational modeling and molecular dynamics simulation of IDPs [294,322]. The latter is one of the few methods that allow for the description of IDPs in their conformational ensemble, instead of a single (or just a few) conformations [132,322,324,333,334,335,336,337].

## 6. Conclusion and Perspectives

The pharmacological strategies developed so far (and reviewed here) that target IDPs can be separated as binding directly to IDPs and hampering their aggregation by keeping them in the interaction incompetent conformation; interacting with the IDP and promoting the stabilization of non-toxic/ non-amyloidogenic oligometric species; and interacting with the amyloidogenic protein and greatly accelerating its aggregation to minimize the period of toxic oligomer formation [338]. Still, as we described here, there are many pathways acting on IDP control, and these are still unexplored targets for pharmaceutical interference. As the binding mechanisms of IDPs are being better described from a physical chemical standpoint [339,340], it is becoming clear that for candidate molecules to act on IDPs they must deviate from traditional prediction rules for drug-likeness [319,320]. One standout feature of IDP ligands is that they are larger and more three-dimensional than traditional drugs [341]. Adding another layer of complexity to this scenario, some proteins are shown to be conditionally unfolded [342], being disordered only under specific conditions. 

Despite being abundant in eukaryotes, in which IDPs have evolutionarily conserved interaction partners [343], the occurrence of disorder in proteins from other organisms is being described. It includes the description of IDPs in Trypanosomatid parasites [344] and in some paramyxoviruses, including measles, Nipah and Hendra viruses [345]. These proteins constitute prospective targets for drug design endeavors, as was also observed for multiple disordered targets in prostate cancer [346]. Furthermore, recent evaluations indicate that IDP-targeted drug development may not be irreconcilable with structure-based drug design [347].

The development of drugs specifically tailored for IDPs is still in its infancy. As with the whole pipeline for drug discovery, there has been continuous progress in this area, and as we proposed in this work, there are many untapped pathways and unexplored targets regarding these proteins. As the biophysical techniques advance to catch up with the diversity of disordered behaviors in proteins, one can expect major developments in this front. Taking the limited but solid cases of success in IDP-specific drug design, we may face a future in which target disorder may be taken as the rule and not the exception.

## Figures and Tables

**Table 1:** Known drugs acting on IDPs (selected examples).

Compound Name *	Targets	Compound Structure
CLR01 (Molecular tweezers)	Lysine and arginine residues in amyloid proteins	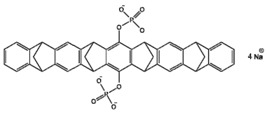
ELN484228	α-Synuclein	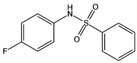
SEN1576	Amyloid β	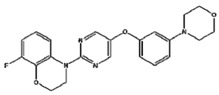
NQTrp	PHF6 (Tau protein)	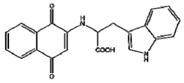
Nutlin-3	p53-MDM2 complex	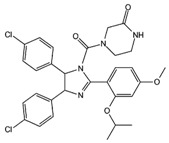
10058-F4	c-Myc-Max complex	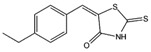
10074-G5	c-Myc-Max complex	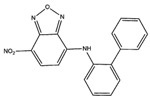
YK-4–279	EWS-Fli1	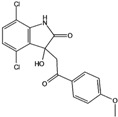
Trodusquemine	PTP1B	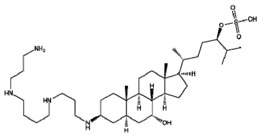

* Unless otherwise specified, these names are directly taken from their lead-compound coding and have no meaning on their own. EWS: Ewing Sarcoma; MDM2: oncoprotein (murine double minute 2); Myc: Myelocytomatosis- transcription factor homolog; NQTrp: naphthoquinone-tryptophan hybrid; PHF6: Tau-derived peptide (amino acid sequence VQIVYK); PTP1B: protein-tyrosine phosphatase 1B.

## Abbreviations

AD	Alzheimer’s Disease
APP	Amyloid Precursor Protein
CJD	Creutzfeldt–Jakob Disease
CNS	Central Nervous System
CS	Conformational Selection
GSS	Gerstmann–Sträussler–Scheinker Disease
IAPP	Islet Amyloid Polypeptide (Amylin)
IDP	Intrinsically Disordered Protein
IDR	Intrinsically Disordered Region
IF	Induced Fit
MT	Microtubules
NMR	Nuclear Magnetic Resonance
PDB	RCSB Protein Databank
PrP	Prion Protein
PrPSc	Prion Protein, Alternate Conformation
PTM	Post-Translational Modification
SLiMs	Short Linear Motifs
ssNMR	Solid-State Nuclear Magnetic Resonance
TAD	Transactivation Domain (of p53)
UD	Ubiquitin-Dependent
UI	Ubiquitin-Independent
